# Research on the impact of the socio-educational environment on the academic performance of college students: the mediating role of study motivation

**DOI:** 10.3389/fpsyg.2023.1289064

**Published:** 2024-01-05

**Authors:** Weiqin Wang, Lu Han, Qingjiao Lu, Xingjun Lv, Yu Liu, Dongxuan Wang

**Affiliations:** ^1^College of Science and Technology, Hebei Agricultural University, Huanghua, Hebei, China; ^2^College of Economics and Management, Hebei Agricultural University, Baoding, Hebei, China; ^3^Department of Basic Courses, Hebei Agricultural University, Huanghua, Hebei, China

**Keywords:** academic performance, data analysis, socio-educational environment, study motivation, questionnaire

## Abstract

**Background:**

Enhancing the academic performance (AP) of college students can contribute to the overall scientific literacy among this population, thereby fostering societal progress.

**Objective:**

The study investigates the correlation between college students’ AP and the socio-educational environment (SEE, including family, roommates, and teachers), study motivation (SM, including self-efficacy and study behaviors). Based on the research findings, recommendations are offered to students, educators, and school administrators.

**Settings:**

Utilizing a stratified sampling approach, data was collected by selecting a sample of 330 first-year computer science students from a specific local university in Hebei Province, China.

**Methods:**

Data will be collected through a hierarchical sampling method. Using correlation analysis, difference analysis, and structural equation modeling (SEM) as data analysis methods. The data passed reliability and validity analysis (*Cronbach’s Alpha* = 0.88, *KMO* = 0.88, *χ^2^*/*df* = 1.49*RMSEA* = 0.04).

**Results:**

The independent sample T-test results showed that female students had higher academic performance than male students (*p* < 0.001), and there was no significant difference in academic performance between students from single parent or orphan families and students from normal families (*p* = 0.14), from non urban areas and from urban areas (*p* = 0.67). The results of the mediating effect analysis indicate that SM exerts complete mediation in the association between SEE and AP, with a mediating effect value of 0.18.

**Conclusion:**

The educational disparity between urban and rural areas in China is gradually narrowing. Support policies for students from impoverished families in higher education institutions are showing initial effectiveness. The conducive learning environment and educational atmosphere for students can indirectly influence their psychological state, thus impacting their academic performance during their university years.

## Introduction

The field of educational studies has gained significant prominence in recent years, serving as a catalyst for scientific and technological advancements worldwide. This exemplifies the indispensable role that education plays in shaping a nation’s future (Feng et al., 2023). During the 20th National Congress of the Communist Party of China in 2022, Chinese President Xi (2022) underscored the imperative of cultivating talents for both the Party and the nation. He stressed the need to comprehensively enhance the caliber of autonomous talent development, with a specific emphasis on nurturing exceptional and pioneering individuals while attracting talents from across the globe. Moreover, in order to achieve the goals of sustainable development, higher education, as a unique entity that encompasses all disciplines, should contemplate how to continuously provide society with high-quality talent (Feng et al., 2023; Gwilliam et al., 2023).

The AP of college students can serve as a predictor of their future achievements in the workplace (Kim, 2020; Peng et al., 2021). It often serves as a comprehensive indicator of students’ learning ability, level of engagement in learning, and proactiveness in their studies. Students with a strong AP can receive scholarships, job offers, and even be recommended to pursue a master’s degree at higher-level universities. This provides a prerequisite for us to study the influencing factors of college students’ AP (Wang et al., 2022). Conversely, students who perform poorly academically may struggle to find ideal employment opportunities and may even be unable to graduate successfully (Zayed et al., 2022). As a result, researchers from different countries are actively conducting theoretical and practical studies to identify factors that impact students’ academic performance and intervene accordingly to improve their academic achievements.

Regarding these influencing factors, we can categorize them into two main aspects: internal factors within the students themselves and external factors that surround the students. For instance, Smith et al. (2023) conducted a study with 302 adolescent participants and found that teenagers with attention deficit or hyperactivity disorders generally exhibit lower levels of learning motivation, which in turn affects their academic performance. The internal factors such as sleep (Boraita et al., 2023; Chan et al., 2023; Hamvai et al., 2023), depression (Song et al., 2023; Urbanska-Grosz et al., 2023), and disordered social media use (Almarzouki et al., 2022; Sserunkuuma et al., 2023) also exert varying degrees of influence on academic performance. In this study, we primarily investigate the internal factors pertaining to learning state, encompassing self-efficacy and learning behavior. Xu et al. (2023) posits that self-efficacy in learning is a significant factor that can influence, and even determine, learning motivation and cognitive emotions. By enhancing self-efficacy in learning, academic performance can be improved. Latino et al. (2023) implemented a physical activity program during breaks to artificially intervene, and found that after 12 weeks, the attention and self-efficacy of most students had significantly improved. Du et al. (2022) believes that students who achieve success in learning follow a learning pattern consisting of “before-class, in-class, and after-class” behaviors, which has been verified. This illustrates that good study habits are key to achieving academic success. From a teacher’s perspective, one of the purposes of changing the teaching mode is to enhance autonomous learning ability. Lu et al. (2023) designed multiple learning activities during actual teaching process and verified this viewpoint.

From an external perspective, there are numerous factors that influence academic performance. In this study, we focus on three aspects: peers, teaching methods employed by teachers, and the family environment, which are the groups with whom students have the most interaction. The phenomenon of peer effect is an inherent law, whereby negative occurrences can spread among companions, such as depressive emotions (Huang et al., 2023). On the contrary, peers can also provide mutual support and assistance in learning, exerting a positive influence that is comparable to that of a teacher in practical activities (Sahin et al., 2023). One of the indicators for evaluating students’ classroom achievements is the assessment of teachers’ teaching. The appropriateness of a teacher’s instructional skills directly impacts students’ classroom gains (Wang et al., 2023). By utilizing advanced teaching equipment, the effectiveness of classroom interaction can be enhanced, allowing students to actively engage in classroom activities (Hu, 2023). By harnessing internet resources, the implementation of flipped classroom pedagogy can break down the constraints of time and space in learning, allowing for a more flexible and accessible approach to education (Ruzafa-Martínez et al., 2023). The influence of the family on academic performance manifests in various aspects. Zhang et al. (2023) showed that family cultural capital, family economic capital, and parental support all contribute to an increased likelihood of becoming a scientist. Conversely, parents’ negative habits can lead to a decline in academic performance. For example, parental anxiety can result in child anxiety (Albulescu et al., 2023), while parents’ smoking habits can influence children to mimic smoking behavior (Zhou et al., 2023), thereby impacting academic performance.

However, how does the social educational environment, including peers, teacher teaching methods, and family, affect AP? Is it related to the SM in individual factors? I suppose that SEE can shape or modify people’s study state and indirectly affect AP. Against this backdrop, this study focuses on computer science students from a local university in Hebei Province, China to explore the relationships SEE, SM, and AP with the aim of analyzing the reasons behind differences in AP. The objective is to analyze the underlying reasons for the differences in academic performance among students from local universities in Hebei Province. This study can explore the impact of social education environment on academic performance, providing suggestions for students, teachers, and educational decision-makers to improve students’ academic performance. For students, it helps to comprehensively develop knowledge and skills, and improve social competitiveness. For teachers, the fundamental task of cultivating morality and nurturing talents can be implemented, the teaching concepts and methods can be continuously improved, therefore, achieving a virtuous cycle of student progress and teacher self-improvement. For educational decision-makers, the plan for further deepening the reform of the education system, promoting the construction of China’s “first-class university,” and cultivating social talents can be achieved.

## Literature review

### Socio-educational environment

The educational environment of students plays a crucial role in influencing their academic performance. As previously mentioned, parents, teachers, and the family are the primary groups that college students interact with on a daily basis. These groups, to a certain extent, represent the majority of individuals students come into contact with during their education, which we refer to as the socio-educational environment (Sahin et al., 2023; Wang et al., 2023; Zhang et al., 2023). The influence of the family on academic performance mainly includes the impact of parental educational methods on children, the effects of family economic capital and social capital on academic success, and the impact of urban–rural educational disparities on academic performance. Urban parents generally have higher levels of education compared to rural parents. This leads to they can invest more abundant time, energy and financial resources in their children’s education. In other words, the higher the parents’ educational attainment is, the more scientifically they educate their children, the greater their academic involvement, and the higher the academic performance their children can achieve. The urban–rural educational disparities primarily manifest in terms of educational resources and teacher qualifications (Zhang et al., 2018, 2020). Compared to urban areas, rural areas lag behind in terms of educational facilities and teachers’ pedagogical approaches. Therefore, these factors have long-term effects on rural children, preventing them from attaining a favorable learning environment (Yuan et al., 2021).

The teaching methods of teachers can also impact students’ AP. The traditional methodology of instruction primarily entails the dissemination of knowledge through theoretical lectures and direct demonstrations during practical laboratory sessions (Deng, 2023). While these methods indeed serve a necessary purpose by directly aiding in students’ comprehension of course content and operational procedures, the integration of novel pedagogical approaches such as online and offline blended teaching methods, can effectively enhance the quality of instruction (Guo et al., 2022).

The improved pedagogical techniques encompass heuristic teaching, discussion-based teaching, problem-based learning, and case-based teaching. Compared to traditional methods, these improved pedagogical techniques are more favorably received by students. They have the ability to captivate students’ learning interests and attentiveness in the classroom, thus enhancing their AP (Zhou et al., 2016). The proactive pedagogical reform by teachers can subtly influence students’ academic resilience, learning motivation, and academic fatigue, thereby indirectly impacting their AP. Teachers who actively explore pedagogical reform and seek progress set a non-academic example for students, fostering intrinsic motivation and providing them with a drive to learn (Trigueros et al., 2020). By proactively enhancing the classroom atmosphere and strengthening interaction with students, teachers significantly improve students’ engagement. It has a positive impact on enhancing students’ AP (Aguilera and Perales-Palacios, 2020; Tong et al., 2022). On the contrary, if teachers fail to assume their rightful responsibilities and resort to violent behavior towards students, it can negatively affect both student behavior and their emotional well-being, ultimately hampering their AP (Kiziltepe et al., 2020).

The Chinese idiom “The environment one is in will have a great influence on one” describes the phenomenon of peer influence or the company one keeps. The phenomenon of peer influence has long been a focal point of research in the fields of education and psychology. A plethora of literature has already demonstrated that peers can exert both positive and negative influences on one another. In the study conducted by Bi et al. (2023) on the impact of depression on academic performance in adolescents, it was found that male students can enhance their academic achievement through the improvement of peer relationships, whereas this improvement was not observed among female students. Ni and Wang (2023) discovered that an increase in the number of friends, regardless of whether they are of the same sex or opposite sex, can lead to a decline in academic performance among Chinese middle school students. Meanwhile, Wu et al. (2023), through a controlled experiment, found that peer feedback can effectively enhance the English academic writing proficiency of Chinese doctors. Similarly, Durak (2022) employed a controlled experimental approach and implemented four different blended teaching methods in classrooms. The research findings indicate that students using peer-assisted methods are more likely to achieve success compared to those using other methods. Additionally, peer assistance has been shown to significantly improve both the learning motivation and self-efficacy within the group, providing favorable evidence for enhancing academic performance. Chinese college students typically spend about 8 months in school each year, and they spend a significant amount of time living with their roommates. Based on previous research findings, we can boldly speculate that the AP of Chinese college students is influenced by their roommates.

### Study motivation

Currently, the scientific community has reached a consensus that learning behavior and learning self-efficacy have a direct impact on academic performance. Learning self-efficacy refers to an emotional state experienced during the learning process. Behavior is influenced by the prevailing emotional state (Kim et al., 2023), and a positive psychological state is more likely to generate positive behaviors. Therefore, in this study, the term “learning state” refers to both learning self-efficacy and learning behavior.

The study behaviors have long been considered a direct factor influencing AP. In their study on “The Relationship Between Improvement in Higher Education Quality and Cognitive, Behavioral, and Personality Factors of Students,” Martinez et al. (2019) found a positive linear relationship between study behaviors and AP. In their research on the impact of academic achievement among secondary school students, del Rosal et al. (2012) employed SEM to identify the optimal factors influencing AP. They found that study behaviors, compared to personal adjustment, exhibited stronger predictive power for AP. Liu (2017) conducted a study focused on vocational education, using multivariable linear regression model analysis. The findings revealed that classroom study behaviors had the greatest impact on academic achievement, followed by extracurricular study behaviors and internship training behaviors. In another similar study conducted by Liu (2016), it was indicated that active note-taking and actively engaging in discussions or seeking advice from teachers are study behaviors that contribute to improved AP.

Indeed, study self-efficacy is also considered a direct factor that influences AP. Since Bandura’s concept of self-efficacy was put forward in 1986, numerous scholars have conducted extensive research in this area. During the investigation of the relationship between self-efficacy, teacher-student relationships, and mathematical performance, Fu (2023) discovered that self-efficacy can serve as a mediating variable. Establishing a positive teacher-student relationship facilitates the enhancement of self-efficacy, reduction of academic anxiety, and improvement in mathematics achievement. Similar research has also been conducted by Ji and Zhao (2021), who employed SEM to reveal that teacher support can indirectly influence academic achievement through its impact on academic self-efficacy. Meng and Zhang (2023) findings in higher education research indicate a significant and positive correlation between academic self-efficacy, academic engagement, and academic achievement. Academic self-efficacy has the capability to directly predict AP.

The existing research findings have some limitations. Firstly, some literature examines the factors influencing AP from a narrow perspective. If intrinsic and extrinsic factors are combined together, it is unclear how they affect AP. Secondly, some literature focuses on preschool or primary/secondary school students as research subjects. Compared to college students, their cognitive development is not yet fully completed. Therefore, the research conclusions may not be entirely applicable to college students. Thirdly, each country has its own national conditions, educational philosophies, and teaching methods, which are not entirely consistent. Therefore, research conclusions may not be universally applicable. Fourthly, students from different levels of universities have varying levels of abilities. For example, in terms of learning ability and study behaviors, there may be no difference between students from Tsinghua University and Peking University, while students from ordinary universities may lag behind those from the aforementioned two universities.

## Method

### Participants and contexts

The participants are students from a provincial university in Hebei Province, China. This university serves as a representative of local general higher education institutions in Hebei Province. In 2022, the test group of participants had a minimum entrance exam score of 515, a maximum score of 537, an average score of 521, and the college entrance examination rank should be at least sixty thousand or so. The research findings of this paper can be applicable to other general higher education institutions in Hebei Province, as well as can be extended to colleges and universities of similar caliber in other provinces nationwide.

### Measures

In order to explore the causal relationship among SEE, psychological factors related to learning, and AP, this study will employ a questionnaire survey method for data collection. The SEE will be assessed based on factors such as family relationships, teaching skills of educators, and roommate relationships. Psychological aspects of learning will be measured through self-efficacy and study behaviors, among other aspects. AP will be evaluated by the research subjects’ grade point averages. To develop the necessary measurement instruments for this study, we will design appropriate questionnaires and conduct comprehensive analyzes by incorporating students’ overall AP from the school’s grading system and survey data on their family backgrounds. These data will contribute to our understanding of the relationship between SEE, psychological factors related to learning, and AP, as well as the extent to which they influence students’ academic achievements.

The dimensions of peers, educators, and family can collectively be referred to as the SEE. Wu and Qian (2007) developed the “Questionnaire on Interpersonal Relationship Quality in University Dormitories.” This questionnaire consists of a total of 19 indicators, divided into four dimensions: emotional harmony among dormitory residents, interpersonal communication in the dormitory, interregional differences in interpersonal dynamics, and disturbances affecting interpersonal relationships in the dormitory. It comprehensively reflects the dormitory relationships among college students. Olson constructed the “Family Adaptability and Cohesion Evaluation Scale II” (FACES II), which is an adapted version of the original scale based on the Chinese family environment. It primarily assesses the aspects of family cohesion and adaptability, examining the family environment. Regarding to SM, it can be observed through learning attitudes and self-efficacy. Tan (2007) developed the “Self-report Scale of Learning Attitude for Primary and Secondary School Students,” which posits that learning attitudes consist of three components: emotional experiences, behavioral tendencies, and cognitive levels. The behavioral tendencies component showcases students’ study behaviors. Xv (2015) developed the “Questionnaire on Learning Attitudes of College Students.” Its dimension of intellectual curiosity includes learning motivation, encompassing cognitive, emotional, and behavioral aspects of learning attitudes. Pintrich and Groot (1990) developed the Academic Self-Efficacy Questionnaire, which divides academic self-efficacy into two dimensions: self-efficacy perception of learning abilities and self-efficacy perception of study behaviors. However, it is worth noting that there is a scarcity of scales and questionnaires targeting teacher-related factors. Upon our observations, it has come to our attention that there is a lack of scales and questionnaires specifically designed for assessing teacher-related factors. As a result, we have taken the initiative to independently design a section pertaining to the evaluation of teaching skills based on practical considerations. Building upon the aforementioned analysis, we aim to develop and refine measurement tools in this regard.

The preliminary development of the questionnaire is divided into two steps. The first step involves reviewing and selecting relevant domestic and international literature and scales, and collecting pertinent information. The second step entails adapting existing mature scales to align with the specific context and requirements. The questionnaire consists of two sections. The first section includes non-scale items, which gather basic information about the students, such as their names, genders, and family backgrounds. The second section comprises test questions that assess five dimensions: peer relationships, teacher influence, family environment, study behaviors, and self-efficacy. Following the completion of this task, we conducted separate discussions with 5 students, 2 frontline teachers, and 1 educational expert. Through these interactions, we made necessary modifications and improvements to the questionnaire, resulting in the final version. The peer scale consists of 5 measurement indicators, the teacher scale comprises 5 indicators, the family relationships scale includes 5 indicators, the study behaviors scale has 3 indicators, and the self-efficacy scale comprises 5 indicators. The whole questionnaire consists of 23 indicators. The scale employs a 5-point Likert scale for scoring. “Extremely Inconsistent” is assigned a score of 1, “Inconsistent” is assigned a score of 2, “Neutral” is assigned a score of 3, “Consistent” is assigned a score of 4, and “Extremely Consistent” is assigned a score of 5.

### Data analysis

The data analysis for this study primarily utilizes SPSS 26.0 software and AMOS 26.0 software. SPSS 26.0 is primarily employed for exploratory factor analysis (EFA) of reliability analysis, correlation analysis, and analysis of variance (ANOVA) in the assessment of validity. On the other hand, AMOS software is primarily utilized for confirmatory factor analysis (CFA) of reliability analysis and constructing a structural equation model (SEM).

## Results

### Data screening

We provided a total of 23 estimated parameters, and calculated the minimum sample size of 230 based on the ratio of 10:1 (sample size: estimated parameter) recommended by relevant researchers (Bentler and Chou, 1987; Jackson, 2003). We have initially gathered 330 questionnaires, resulting in a sample size of 330 for the study, which exceeds the minimum requirement of 230. In order to ensure the questionnaires possess sufficient reliability, we have excluded the 15 questionnaires that were completed in the shortest amount of time. Among the remaining 315 questionnaires, no instances of missing values or outliers have been identified. Therefore, there are a total of 315 valid samples in the final dataset.

### Reliability and validity analysis

#### Reliability analysis

The reliability test results are presented in Table 1. The overall reliability is 0.88, with a reliability of 0.83 for peer influence, 0.90 for teacher influence, 0.90 for family influence, 0.83 for self-efficacy influence, and 0.77 for study behaviors. The results of the credibility test are shown in Table 1. The overall reliability coefficient is 0.88, with a reliability coefficient of 0.83 for the peer section, 0.90 for the teacher section, 0.90 for the family section, 0.83 for the self-efficacy section, and 0.77 for the study behavior section. With the exception of the slightly lower reliability in the dimension of study behaviors, all other dimensions have achieved a reliability of 0.80 or above. This indicates that the questionnaire has good reliability and can be processed to the next step of analysis.

**Table 1:** The reliability test.

	Cronbach’s Alpha	Number of indicators
Roommate	0.83	5
Teacher	0.90	5
Family	0.90	5
Study self-efficacy	0.83	5
Study behavior	0.77	3
Total	0.88	23

#### Validity analysis

The validity analysis is mainly conducted through factor analysis. Factor analysis can be divided into exploratory factor analysis (EFA) and confirmatory factor analysis (CFA).

**Figure 1:**
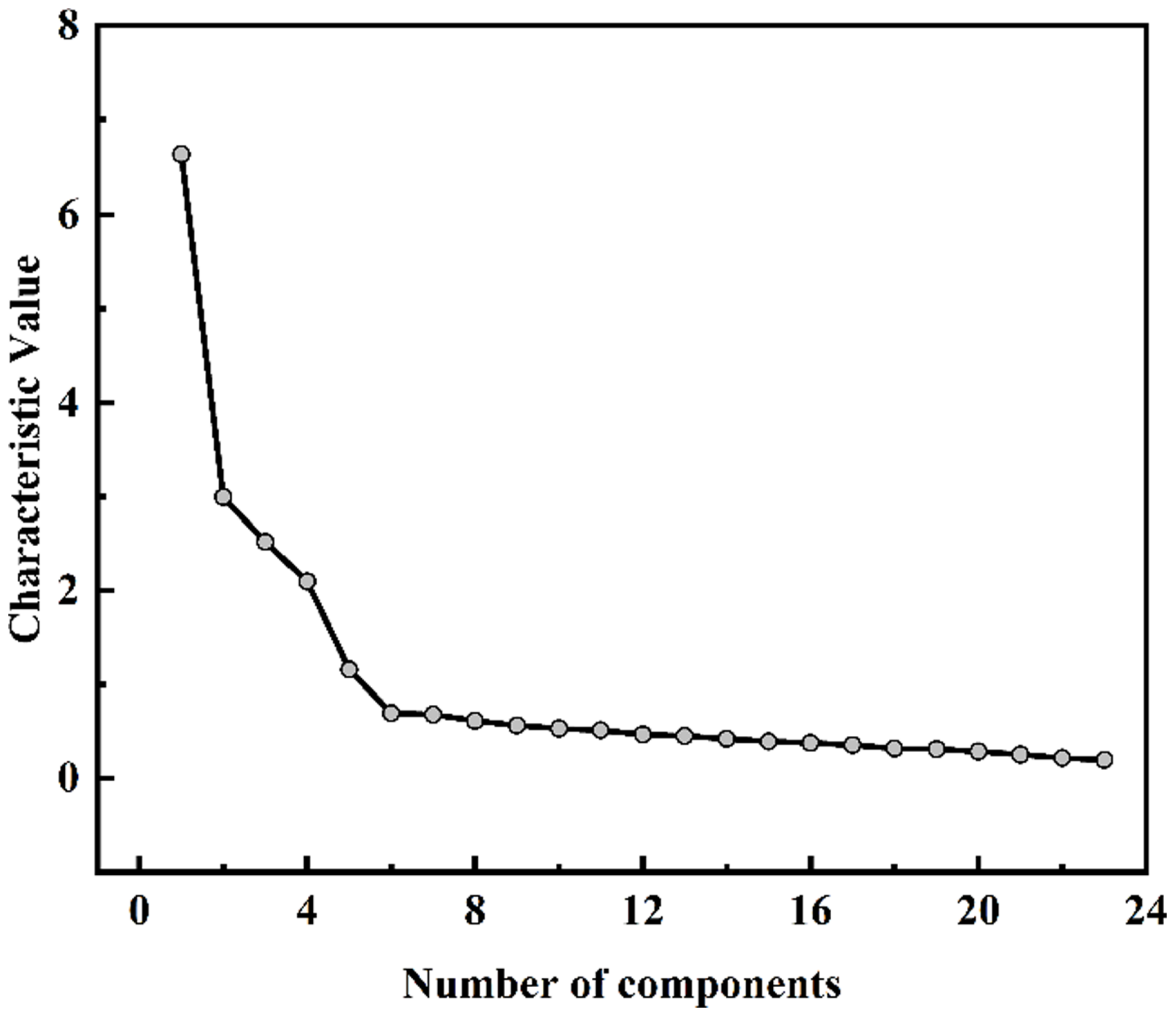
The scree plot.

Perform KMO and Bartlett’s sphericity test on a sample of 315 survey responses. Upon examination the overall questionnaire *KMO* = 0.88, *χ^2^* = 3635.70, *df* = 253, *p* < 0.05.The test results demonstrate the fulfillment of the prerequisites for EFA. In the results of principal component analysis, there are five factors with eigenvalues greater than 1 (as shown in Table 2, only the factors with eigenvalues greater than 0.60 were selected). In the scree plot, the first five factors exhibit steep slopes, while the slope becomes gentler starting from the sixth factor (as illustrated in Figure 1). Based on the eigenvalues and the scree plot, it is advisable to select five dimensions, which aligns with the initial assumptions. By computing the component matrix of the 23 indicators after rotation (as shown in Table 3) and observing the factor loading coefficients of each item on the five dimensions, it can be concluded that the indicators are consistent with the predetermined dimensions. Furthermore, it can be observed from Table 2 that the cumulative variance contribution rate of the five factors reaches 66.99%, with the largest factor variance contribution rate being 15.67%. Therefore, it can be concluded that there is no significant issue of common method bias in the questionnaire data.

**Table 2:** Total variance exposition.

	Initial eigenvalue			Sum of extracted load squares			Sum of rotational load squares		
	Total	Variance	Cumulative variance %	Total	Variance	Cumulative variance %	Total	Variance	Cumulative variance %
1	6.65	28.89	28.89	6.65	28.89	28.89	3.60	15.67	15.67
2	3.00	13.02	41.91	3.00	13.02	41.91	3.60	15.66	31.32
3	2.52	10.95	52.86	2.52	10.95	52.86	3.16	13.73	45.05
4	2.10	9.11	61.97	2.10	9.11	61.97	3.10	13.48	58.53
5	1.16	5.02	66.99	1.16	5.02	66.99	1.95	8.46	66.99
6	0.69	3.01	70.00						
7	0.68	2.94	72.94						
8	0.61	2.65	75.59						

**Table 3:** The component matrix after rotation.

Indicators	Component				
	1	2	3	4	5
Family Relations1		0.87			
Family Relations2		0.85			
Family Relations3		0.82			
Family Relations4		0.80			
Family Relations5		0.75			
Teacher’s Teaching Skills1	0.85				
Teacher’s Teaching Skills2	0.84				
Teacher’s Teaching Skills3	0.81				
Teacher’s Teaching Skills4	0.78				
Teacher’s Teaching Skills5	0.78				
Roommate Relations1				0.81	
Roommate Relations2				0.75	
Roommate Relations3				0.77	
Roommate Relations4				0.74	
Roommate Relations5				0.72	
Study Behavior1			0.83		
Study Behavior2			0.72		
Study Behavior3			0.68		
Study Self-Efficacy1					0.72
Study Self-Efficacy2					0.69
Study Self-Efficacy3					0.78
Study Self-Efficacy4					0.80
Study Self-Efficacy5					0.79

The dataset consisting of 315 questionnaires was subjected to a validity assessment using the AMOS 26.0 software, specifically employed for CFA. The validity analysis can be conducted from three perspectives: construct validity, convergent validity, and discriminant validity. These aspects will be examined to ensure the robustness of the measures. The validity analysis of the results showed that the chi square ratio of degrees of freedom and RMSEA were, respectively, 1.65 and 0.05 and the NFI, RFI, IFI, TLI, and CFI are, respectively, 0.91, 0.90, 0.96, 0.95, and 0.96, which met the requirements (as shown in Table 4). All indicators have reached excellent levels, indicating that the questionnaire has good construct validity. The convergent validity analysis result is depicted in Table 5, where the Average Variance Extracted (AVE) of each dimension ranges from 0.51 to 0.65, and the Composite Reliability (CR) ranges from 0.77 to 0.90. This indicates that the questionnaire has good convergent validity. The discriminant validity analysis result is depicted in Table 6, where the square root of the AVE for each factor is greater than the maximum correlation coefficient between that factor and other factors. This indicates that the questionnaire has good discriminant validity.

**Table 4:** The construct validity analysis.

	Result	Standard
*χ*^2^/df	1.65	<3.00
RMSEA	0.05	<0.08
Normed fit index(NFI)	0.91	>0.90
Relative fit index(RFI)	0.90	>0.90
Incremental fit index (IFI)	0.96	>0.90
Tucker-Lewis index(TLI)	0.95	>0.90
Comparative fit index (CFI)	0.96	>0.90

**Table 5:** The convergent validity analysis.

	Path	Standardized estimate	AVE	CR
Family F1	F1 → Family Relations1	0.89	0.64	0.90
	F1 → Family Relations2	0.84		
	F1 → Family Relations3	0.77		
	F1 → Family Relations4	0.77		
	F1 → Family Relations5	0.74		
Teacher F2	F2 → Teacher’s Teaching Skills1	0.73	0.65	0.90
	F2 → Teacher’s Teaching Skills1	0.77		
	F2 → Teacher’s Teaching Skills1	0.78		
	F2 → Teacher’s Teaching Skills1	0.87		
	F2 → Teacher’s Teaching Skills1	0.85		
Roommate F3	F3 → Roommate Relations1	0.78	0.51	0.84
	F3 → Roommate Relations1	0.71		
	F3 → Roommate Relations1	0.70		
	F3 → Roommate Relations1	0.73		
	F3 → Roommate Relations1	0.64		
Study Behavior F4	F4 → Study Behavior1	0.74	0.52	0.77
	F4 → Study Behavior2	0.74		
	F4 → Study Behavior3	0.69		
Study Self-Efficacy F5	F5 → Study Self-Efficacy1	0.75	0.54	0.85
	F5 → Study Self-Efficacy2	0.80		
	F5 → Study Self-Efficacy3	0.68		
	F5 → Study Self-Efficacy4	0.68		
	F5 → Study Self-Efficacy5	0.75		

AVE represents the average variance extracted, CR represents composite reliability.

**Table 6:** The discriminant validity analysis.

	Study self-efficacy	Study behavior	Roommate	Teacher	Family
Study self-efficacy	0.73				
Study behavior	0.63	0.72			
Roommate	0.14	0.30	0.71		
Teacher	0.30	0.41	0.37	0.80	
Family	0.27	0.40	0.33	0.33	0.80
AVE	0.54	0.52	0.51	0.65	0.64

#### Correlation analysis

Utilizing the SPSS 26.0 software, we shall conduct a correlation analysis among the 315 questionnaire responses across five dimensions, namely peers, teachers, family, study behavior, and study self-efficacy. The results are presented in Table 7. Except the relationship between roommate relations and AP is not significant; all other relationships demonstrate significant correlations.

**Table 7:** Pearson correlation analysis.

	Roommate relations	Teacher’s teaching skills	Family relations	Study behavior	Study self-efficacy	AP
Roommate Relations	1					
Teacher’s teaching skills	0.33**	1				
Family relations	0.30**	0.31**	1			
Study behavior	0.24**	0.34**	0.33**	1		
Study self-efficacy	0.17*	0.25**	0.23**	0.51**	1	
AP	0.08	0.14*	0.22**	0.29**	0.21**	1
*M*	3.70	4.03	3.85	2.93	3.34	0.03
SD	0.76	0.70	0.85	0.79	0.67	0.92

*p < 0.05, **p < 0.01, *M* represents the mean, SD represents standard deviation.

#### Differential analysis

In order to investigate the differences in AP among students based on gender, family background, and educational area, an independent samples t-test was conducted. The results are presented in Table 8. The normality test revealed that AP follows a normal distribution (*p* < 0.001), which satisfies the assumptions for conducting an independent samples *t*-test.

**Table 8:** The independent samples T-test.

Grouping variables		*M ±* SD	*t*	*p*
Gender	Male (*n* = 214)	−0.12 ± 1.00	−4.11	0.00
	Female (*n* = 101)	0.33 ± 0.62		
Family	Normal family (*n* = 282)	−0.00 ± 0.92	−1.50	0.14
	Special family (*n* = 33)	0.25 ± 0.89		
Educational area	Non-urban area (*n* = 251)	0.37 ± 0.91	0.42	0.67
	Urban area (*n* = 64)	−0.02 ± 0.95		

Based on the results of the independent samples t-test, it was found that male students have lower AP compared to female students. This difference is statistically significant at a significance level of 1%, indicating a significant gender difference in AP. According to the analysis results, there is no significant difference in AP based on whether or not there are special circumstances within the family. Special circumstances refer to situations such as economic difficulties, single-parent households, or being an orphan. Based on previous research findings, urban students tend to have higher AP compared to non-urban students due to factors such as abundant educational resources. In this study, students’ areas of residence were categorized into urban and non-urban area. However, the analysis results indicate that there is no significant difference in AP based on whether students receive education in urban or non-urban areas.

#### Mediation analysis

##### Preliminary SEM

**Figure 2:**
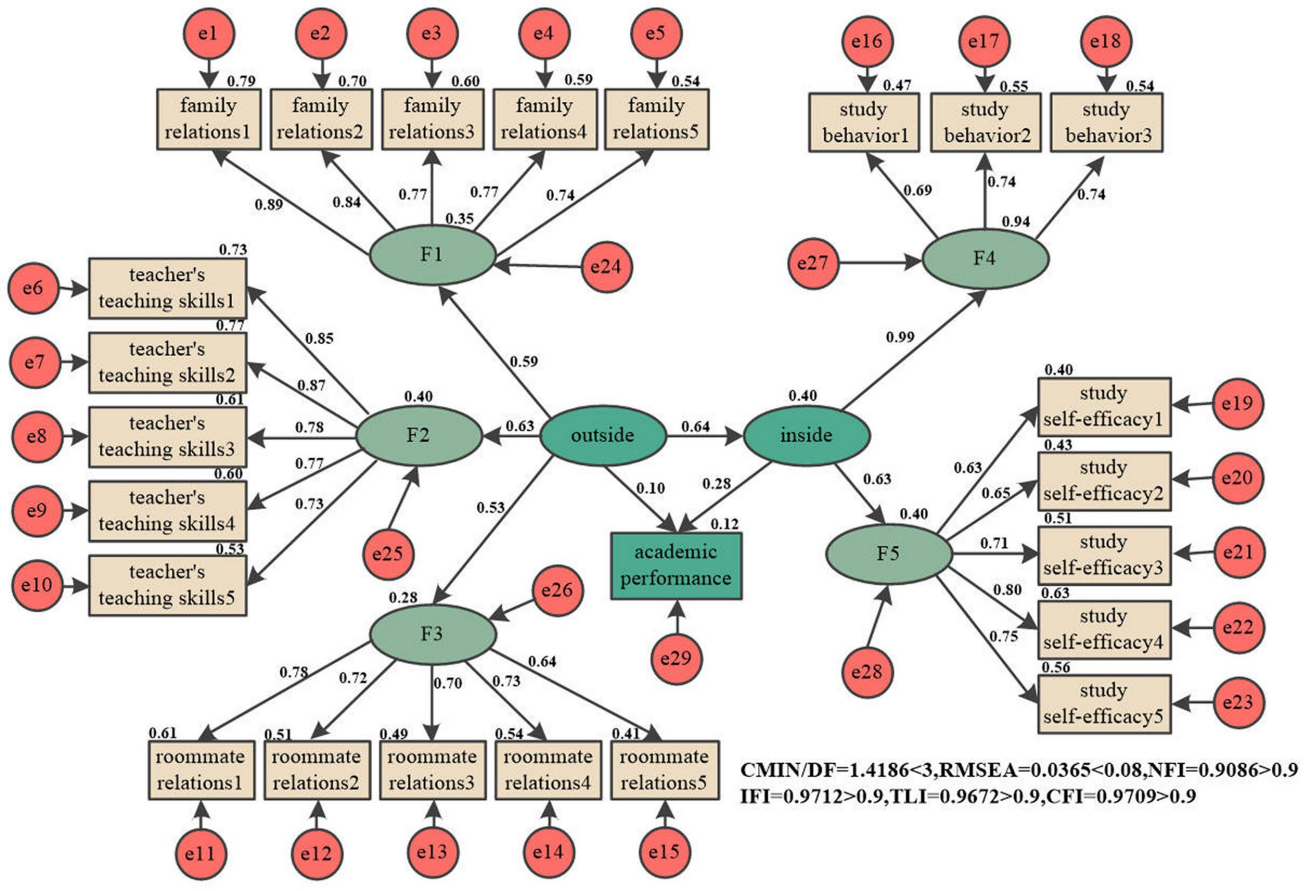
The preliminary SEM.

Applying a second-order SEM to assess the relationships among SEE, SM and AP, as illustrated in Figure 2. Table 9 presents the results of the bootstrap analysis on the measurement instrument, providing standardized and non-standardized path coefficients, total effects, direct and indirect effects, significance of non-standardized path coefficients, and 95% confidence intervals. The observed variables for the SEE include peers, teachers, and family, with loadings ranging from 0.53 to 0.63. Regarding the observed variables for SM, they consist of study behavior and self-efficacy, with loadings ranging from 0.63 to 0.99. The path coefficient between the SEE and SM is 0.64 (*p* < 0.01), with the 95% confidence interval not containing zero, indicating a significant positive influence. The path coefficient between SM and AP is 0.28 (*p* < 0.01), with the 95% confidence interval not containing zero, indicating a significant positive influence. However, the path coefficient between the SEE and AP is 0.10 (*p* > 0.05), with the 95% confidence interval containing zero, suggesting that there is no significant positive influence.

**Table 9:** The preliminary SEM Bootstrap test.

	Path	Standard error	Effect size	Lower	Upper	*p*
Direct effect	SEE→SM	0.08	0.64	0.48	0.80	0.00
	SM → AP	0.13	0.28	0.05	0.54	0.02
	SEE→AP	0.13	0.10	−0.18	0.34	0.46
Indirect effect	SEE→SM → AP	0.09	0.18	0.04	0.41	0.02

##### Improved SEM

**Figure 3:**
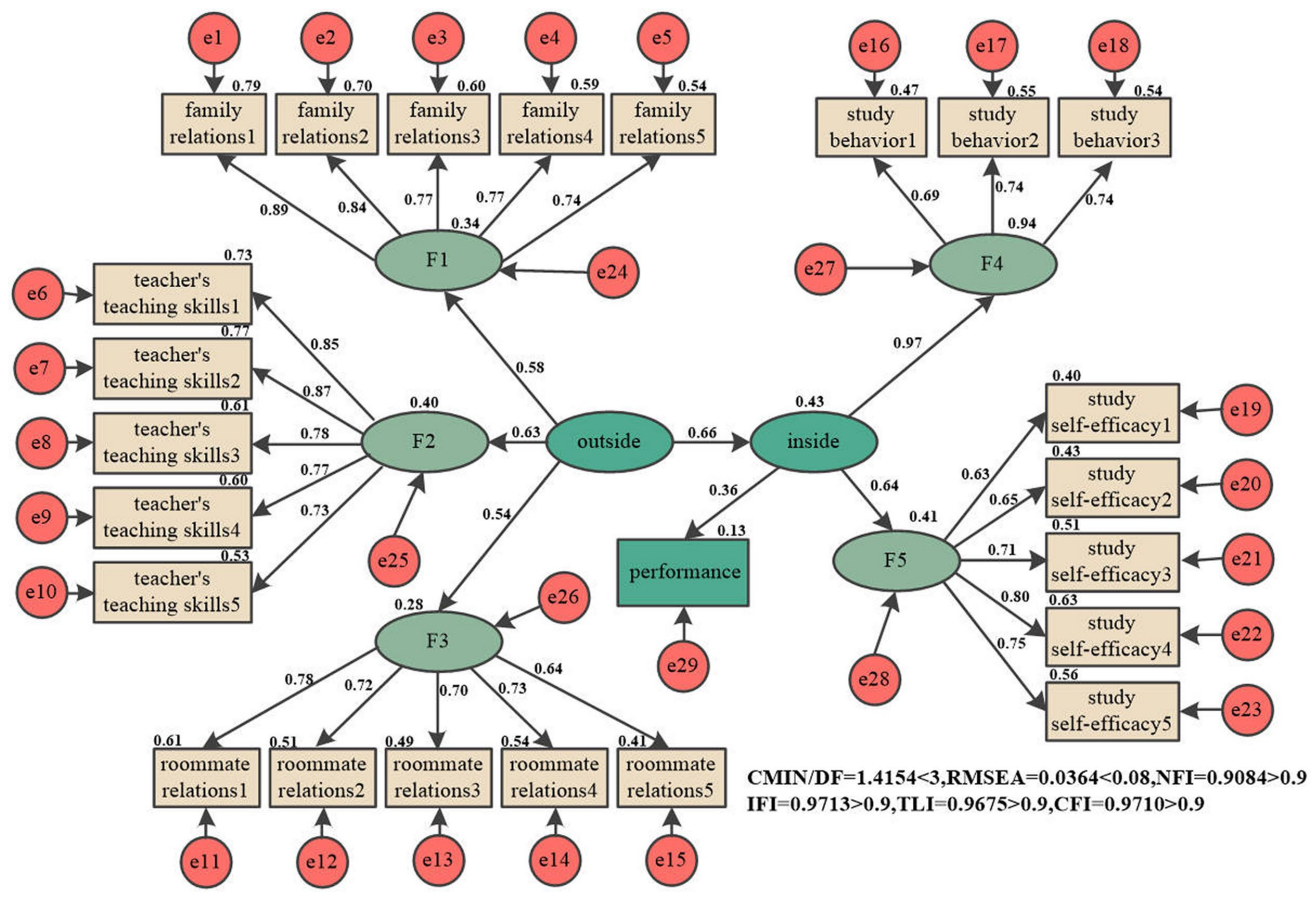
The revised SEM.

Based on the preliminary analysis of the SEM, we speculate that there is a complete mediating effect between the SEE, psychological factors related to learning, and AP. Specifically, the SEE indirectly influences AP by affecting SM. Therefore, we revised the model by removing the direct influence of the SEE on AP. The results of the structural equation model testing are presented in Figure 3, while the Bootstrap test results are shown in Table 10. After removing the path connecting the SEE and AP, the impact of SM on AP remains significant. The indirect effect of the SEE on AP through its influence on SM is estimated to be 0.18.After the revision, the model fit indices demonstrate a satisfactory level of fit. The *χ*^2^/*df* value is 1.42 (<3), *RMSEA* is 0.04 (<0.08), and NFI, IFI, TLI, and CFI are all greater than 0.90. These results indicate that the revised model has reached a desirable level of fit.

**Table 10:** The revised SEM Bootstrap test.

	Path	Standard error	Effect size	Lower	Upper	*p*
Direct effect	SEE→SM	0.08	0.66	0.49	0.81	0.00
	SM → AP	0.07	0.36	0.21	0.50	0.00
Indirect effect	SEE→SM → AP	0.06	0.23	0.12	0.36	0.00

## Discussion

### Discussion of differential analysis results

In terms of gender differences, male students exhibit lower academic performance than their female counterparts. This finding contradicts our traditional beliefs. Based on previous research analysis, males have been found to exhibit stronger superiority in logical thinking and have a higher working memory capacity (Pezzuti et al., 2020). Therefore, during the primary and secondary school stage, there does exist a phenomenon where male students demonstrate superior scientific reasoning ability and academic performance compared to female students (Luo et al., 2021). When it comes to studying computer science or other technical fields, it is often assumed that males are naturally more skilled than females. However, research has found that the gender-based differences in these areas are unstable and vary across different research contexts (Vo and Csapó, 2020). In the field of computer programming, Yang and Bers (2023) conducted an observational study on the manifestation of programming skills in early childhood and found that there were no significant differences in programming abilities between boys and girls. Therefore, we can infer that gender does not necessarily lead to differences in logical thinking among individuals. By analyzing the research findings on engineering education in the past 2 years, we have discovered that female students do not perform worse than male students academically. Tong (2005) conducted a statistical analysis and did not find any evidence suggesting that males have an advantage in STEM (Science, Technology, Engineering, and Mathematics) subjects. On the contrary, females have shown superior performance in multiple mathematics-related courses compared to males. Wrigley-Asante et al. (2023) conducted a comparative study between college students’ performance in STEM subjects in Advanced Placement (AP) exams and their high school subject grades. They found that male students had better AP scores during their high school years. However, during their college years, female students showed an improvement in their AP scores compared to male students. They argue that it is necessary to break the stereotypes associated with STEM subjects, as female students demonstrate greater levels of dedication, self-discipline, and commitment to learning compared to male students. Although male students may have advantages over female students in engineering job opportunities, female students strive to improve their competitiveness to ensure good job prospects after graduation, leading to differences in their learning styles (Chu, 2017). As a result, it is observed that female engineering students perform significantly better than male students in major coursework. Our research findings are consistent with those of the aforementioned scholars. Although male students may initially have an advantage in computer learning, they tend to be overconfident in their abilities, which can reduce their motivation and enthusiasm for learning. On the other hand, although female students may face disadvantages in computer-related fields, they often strive to compensate for these disadvantages through diligent study. Furthermore, an additional possibility exists whereby the presence of stereotypes poses a threat, as it implies that gender-based irrational beliefs can have a detrimental impact on the intellectual performance of women (Takatsuka et al., 2022). As a result, male students exhibit a more favorable attitude towards learning programming in comparison to their female counterparts (Sun et al., 2022). However, it is important to note that this study primarily focuses on first-year students enrolled in foundational courses, where computer programming classes are rarely included in the curriculum. Additionally, the increased dedication of female students to their academic pursuits exacerbates the widening gap between genders.

The detrimental familial environment appears to exert no discernible impact on students’ academic performance. The findings of this study are inconsistent with those of other scholars’ researches. Regarding students from financially disadvantaged backgrounds, other scholars’ researches tend to suggest that their academic performance will significantly lag behind those who come from relatively high-income families. Poverty exerts a direct negative impact on students’ academic achievement, and increasing family income is fundamental to ensuring children’s academic success (Li et al., 2023). Scholar Wang (2017) revealed the significant impact of family economic capital on college students’ academic performance. Similarly, Li and Ou (2018) found that the disposable income of impoverished families can also have a certain influence on academic achievement. For students with more unique family structures such as single-parent or left-behind children, other scholars also suggest that academic performance of students from single-parent or reconstituted families is worse than that of students from two-parent families (Dufur et al., 2023). Scholar Suk (2021) conducted research and found that the level of single-parent children in CSAT (College Scholastic Ability Test) scores, family education support, school education experience, and student education experience is lower than that of two-parent children. As per these research findings, the varying social economic conditions within households can lead to differences in students’ educational investment, educational impact, psychological influence, and ultimately affect their academic performance. However, the findings of this study are more congruous with the actual circumstances of the research subjects. Currently, in accordance with relevant policies established by the Chinese government and educational institutions, students from disadvantaged family backgrounds are receiving academic support. For instance, subsidies are being provided to impoverished students as well as their families. Furthermore, national scholarships, institutional grants, national student loans, and part-time job opportunities are offered to alleviate financial burdens. As a result, students from financially stable backgrounds and those from disadvantaged family backgrounds do not exhibit significant disparities in terms of economic conditions. Moreover, students from underprivileged families demonstrate greater determination to achieve outstanding academic results by persisting through adversity. Considering that university students are of legal age and exhibit higher levels of psychological maturity, these factors may contribute to minimizing the influence of their family backgrounds on their academic performance (Götz et al., 2020; Slobodskaya and Kornienko, 2021).

In terms of educational resources, there is no significant disparity in academic performance between students from urban and rural areas. It is widely acknowledged that there does exist a developmental gap between urban and rural regions. It cannot be denied that students receiving education in urban areas have access to more comprehensive educational resources compared to their rural counterparts. This disparity is attributable to the differential pace of development between urban and rural areas, which is understandable. In recent years, China’s education policy has gradually shifted towards prioritizing rural areas. Various levels of government have increased investment in rural basic education, indicating a growing focus on rural areas in educational policies. For example, the government has increased financial investment to improve rural infrastructure conditions. Additionally, there is a recognition of the cognitive and teacher quality disparities between urban and rural areas, emphasizing the priority of improving the quality of rural teachers to promote educational equity (Zheng et al., 2023). Therefore, China employs various methods such as recruitment, training, and providing financial support to attract excellent teachers to teach in rural schools. Furthermore, with the development of the digital economy (Jiang et al., 2022), digital learning resources and distance education services are being provided (Zhang et al., 2017) to compensate for the relative lack of educational resources in rural schools. This series of measures has contributed to the gradual reduction of the urban–rural education resource gap. Lastly, the efforts made by the students themselves are also crucial (Asadullah et al., 2021).

### Discussion of correlation analysis results

The correlation between learning behaviors and self-efficacy is significantly positive. This signifies that the more proactive one’s learning behaviors are, the more knowledge and skills they acquire, thereby enhancing their self-efficacy. In other words, possessing higher self-efficacy can stimulate more proactive learning behaviors. This finding aligns with the research findings of other scholars. The research conducted by Hammad et al. (2022) confirms a significant positive correlation between students’ self-efficacy and academic achievement, as well as between learning motivation and academic achievement. Furthermore, in other studies, it has been found that self-efficacy can influence learning behaviors both directly and indirectly through its impact on intermediate variables. For instance, the academic self-efficacy of college students indirectly affects their learning behaviors by influencing their learning motivation (Bai et al., 2020). Similarly, self-efficacy itself can act as an intermediate variable affecting learning behaviors. Zhang and Xv (2023) not only confirmed a significant positive correlation between self-efficacy and learning behaviors among college students but also discovered that place identity indirectly influences learning behaviors through self-efficacy. In other words, self-efficacy plays a partial mediating role in the relationship between place identity and learning behaviors.

The relationship between roommate dynamics and learning behaviors exhibits a significant positive correlation. This finding aligns with the aforementioned phenomenon of peer effects. Within the university context, students spend a considerable amount of time with their roommates, and their behaviors, including their study habits, can mutually influence one another. Certain students are more strongly influenced by peer effects, specifically those with a higher degree of peer attachment. These individuals tend to demonstrate better adaptation and conformity (Xu and Tu, 2023), resulting in a higher consistency between their learning behaviors and those of their peers. Kaehwan (2020) have found through their research that peer attachment significantly influences intrinsic learning motivation. Additionally, peer attachment exhibits a partial mediating effect between self-esteem and intrinsic learning motivation. Since learning motivation has a significant positive impact on autonomous learning behaviors (Bai et al., 2020), it can also confirm the significant positive correlation between peer effects and learning behaviors. However, the correlation between roommate dynamics and learning behaviors tends to weaken gradually over time due to cumulative factors. Cheng and Liang (2022) conducted research revealing that dormitory peers have a significant positive impact on individuals’ engagement in negative classroom behaviors, positive classroom behaviors, and negative extracurricular learning behaviors. However, as students make progress in their academic pursuits, their social circles expand, which makes them spend less time with their roommates. Consequently, this influence tends to gradually diminish over time.

There is a significant positive correlation between a teacher’s teaching skills and students’ learning behaviors. This result suggests that students’ learning behaviors are easily influenced by the teacher’s teaching methods. The stronger a teacher’s teaching ability is, the more proactive the students’ learning behaviors become. This is consistent with our understanding. Xie et al. (2017) research demonstrated that six types of teaching behaviors, such as “clearly explaining course objectives and requirements,” have a significant impact on students’ learning behaviors. Li and Xue (2023) study also confirmed that positive teacher behaviors are moderately correlated with students’ participation in classroom learning.

The correlation between family relationships and academic performance is significantly positive. The household serves as the developmental environment for every student, as well as the breeding ground for cultivating habits. Harmonious family relationships contribute to the cultivation of students’ good learning behaviors, while parenting styles have a positive impact on students’ self-efficacy (Chen et al., 2023). The environmental factors within the family also exert a considerable influence on academic achievements (Ortiz-de-Villate et al., 2021). This viewpoint has also been substantiated by scholars such as Otero et al. (2021), who affirm that family control and support influence students’ academic performance. Similarly, Cheng et al. (2012) has reached the conclusion that family support indeed correlates with students’ success in university studies. Beyond subtly shaping students’ study habits, the family environment also impacts academic performance by influencing students’ learning states. Research conducted by Wang (2020) reveals a significant relationship between family environmental factors and students’ test anxiety. In other words, certain family factors contribute to the occurrence of test anxiety and other related issues, which ultimately affect students’ final academic performance.

The correlation between teaching skills and academic performance is significantly positive. Factors such as teachers’ knowledge base, teaching methods, and teaching skills directly impact students’ academic achievement. This conclusion has been validated by numerous scholars and has become the mainstream view (Aguilera and Perales-Palacios, 2020; Tong et al., 2022). Tao et al. (2022) also proved that the impact of teacher support and its three dimensions on students’ academic performance is significant. Similarly, these teaching skills of teachers can also affect students’ learning motivation, problem-solving ability, and ultimately academic performance, thus having a positive impact on academic achievements (Retnawati, 2022).

There is no significant correlation between roommate relationships and AP. This indicates that there is no direct relationship between the two, but it does not mean there is no indirect relationship. Roommates can indirectly influence AP through the mutual impact on study behaviors. This viewpoint has also been demonstrated in SEM. Scholar Ha (2016) conducted research on the allocation system of university dormitories in China and the influence of roommates. It was found that roommate selection and behavior significantly affect students. Students’ mimicking behavior can lead to changes in their study behaviors, thereby affecting AP.

### Discussion of SEM results

At present, numerous scholars have provided evidence that the environment in which an individual is situated can influence their behavior and attitudes. For instance, the work milieu can exert an influence on psychological capital and innovative conduct (Lee et al., 2022). The perceived neighborhood environment holds the potential to impact behaviors such as sedentary lifestyles, smoking, and alcohol consumption (Liu et al., 2022). Furthermore, street conditions can influence pedestrian route selection (Jin et al., 2022), while the clinical practice environment can influence innovative behaviors. Similarly, this study confirms the impact of the educational environment on one’s SM. Analysis of the results obtained through structural equation modeling reveals that the educational environment influences the state of learning, subsequently influencing academic performance. The influence of roommate relationships on learning disposition is primarily attributed to the presence of peer effects. Peers play a crucial role, either positively or negatively, in various aspects of college students’ lives (Madtha et al., 2023). Peer effects have been proven to impact students’ attitudes (Poquet and Assoc Comp, 2021). However, the outcomes resulting from peer effects vary from person to person. If an individual has a strong attachment to their peers, they tend to exhibit better adaptability and compliance in a new environment. In other words, their learning disposition is more influenced by their peers, and their learning disposition becomes more similar to that of their peers (Xu and Tu, 2023). Teachers play a significant role in influencing students’ learning disposition, which is mainly manifested through their teaching skills, teaching habits, and teacher-student relationships. Over an extended period of interaction with teachers, their behavior and habits can subtly impact students (Yang and Kaiser, 2022). Teachers with excellent teaching skills can make learning easier for students and improve their learning disposition. Additionally, teachers with good teaching habits can help regulate students’ learning disposition (Beausaert et al., 2013). High-quality teacher-student relationships also have significant benefits for students (Poling et al., 2022; Sun and Shi, 2022). The family environment is where individuals develop their behavioral habits, making family education crucial (Volodina, 2022). Therefore, family relationships directly influence students’ learning disposition (Manukaram and Abdullah, 2021). From this, it can be deduced that the educational environment has a significant impact on students’ study states. Drawing upon Burrhus Frederic Skinner’s behaviorism theory and Albert Bandura’s social learning theory, we postulate that learning behavior changes in response to changes in learning outcomes. Positive outcomes reinforce learning behavior, while negative outcomes weaken it, thereby establishing a feedback loop (Yin, 2010). Vu et al. (2022) referred to this cycle as a positive feedback loop and provided an apt description: motivation feeds achievement, and achievement in turn motivates, creating a mutually beneficial causal relationship. This also explains the close connection between learning status and academic performance.

### Limitations

The teacher dimension in the questionnaire is assessed and filled in by students, which can introduce a certain degree of subjectivity. The teaching methods of the same teacher are easy for some students to accept, while others are not.

The AP data utilized in this study was generated during the period of the COVID-19 pandemic. During the pandemic, there was a slight relaxation in course management, with assessment methods primarily focusing on open-book examinations, course projects, and research papers. Although these assessment methods hold some reference value, they cannot fully capture students’ AP compared to closed-book examinations.

Regarding external factors, beyond peers, teachers, and family, there may exist additional potential factors within the learning and living environment of students that can impact their study behaviors and self-efficacy. For different universities, the management style and academic atmosphere of the institution may also influence students’ study behaviors. Similarly, there may be other mediating variables that can impact students’ AP, such as study strategy. These aspects will be the focus of our future research.

## Conclusion

This study investigated the factors influencing AP among 315 students by using analysis of variance, correlation analysis, and SEM. The findings revealed that the urban–rural disparity in China is gradually decreasing. Furthermore, female students are demonstrated better AP compared to male students. The SEE was found to have a fully mediating role in both SM and AP. Based on these research results, several recommendations are proposed for students, teachers, and school administrators.

Students should engage in more communication with peers who excel beyond their own capabilities and learn from their lifestyle and study methods. When it comes to studying, one should remain humble in victory and resilient in defeat. Teachers should proactively enhance their research capabilities and set an example for their students. Utilizing holiday periods to engage in learning exchanges, they should absorb advanced teaching methods and pedagogical concepts, actively seek educational reforms, integrate information technology into the classroom to cultivate students’ interest in learning. Teachers need to strengthen interventions concerning students’ study behavior and AP. Timely recognition should be given to outstanding students, while encouragement and assistance should be provided to those who are struggling, thereby creating a virtuous cycle. School administrators should proactively seek to understand the family situations of each student and provide assistance to those facing economic hardships. They should establish frequent communication with students’ parents to stay informed about their academic progress and daily lives. When assigning student dormitories, it is advisable to avoid concentrating students with poor AP in one dormitory, aiming to prevent the formation of a negative cycle.

## Data availability statement

The original contributions presented in the study are included in the article/Supplementary material, further inquiries can be directed to the corresponding authors.

## Ethics statement

The studies involving humans were approved by Agricultural University of Hebei. The studies were conducted in accordance with the local legislation and institutional requirements. The participants provided their written informed consent to participate in this study.

## Author contributions

WW: Writing – original draft, Writing – review & editing. LH: Writing – original draft, Writing – review & editing. QL: Writing – original draft, Writing – review & editing. XL: Writing – original draft, Writing – review & editing. YL: Writing – original draft, Writing – review & editing. DW: Writing – original draft, Writing – review & editing.
